# Supplementary material for: Dual-gated single-molecule field-effect transistors beyond Moore’s law
Source: Nat Commun. 2022 Mar 17;13:1410. doi: 10.1038/s41467-022-28999-x (PMC8931007; doi:10.1038/s41467-022-28999-x)
Supplement: Supplementary file 1 — Supplementary Information [file 41467_2022_28999_MOESM1_ESM.pdf]

Supplementary Information for

Dual-Gated Single-Molecule Field-Effect Transistors beyond Moore's Law

Linan Meng[1,2†], Na Xin[1†], Chen Hu[3†], Hassan Al Sabea[4†], Miao Zhang[6†], Hongyu Jiang[2,5], Yiru Ji[2,5], Chuancheng Jia[6], Zhuang Yan[1], Qinghua Zhang[2], Lin Gu[2], Xiaoyan He[4], Pramila Selvanathan[4], Lucie Norel[4], Stéphane Rigaut[4]*, Hong Guo[3]*, Sheng Meng[2,5]* and Xuefeng Guo[1,6]*

[1]Beijing National Laboratory for Molecular Sciences, National Biomedical Imaging Center, College of Chemistry and Molecular Engineering, Peking University, 292 Chengfu Road, Haidian District, Beijing 100871, P. R. China.

[2]Beijing National Laboratory for Condensed Matter Physics, Institute of Physics, Chinese Academy of Sciences, Beijing 100190, P. R. China.

[3]Center for the Physics of Materials and Department of Physics, McGill University, Montreal, Quebec H3A 2T8, Canada.

[4]Univ Rennes, CNRS, ISCR (Institut des Sciences Chimiques de Rennes)-UMR 6226, F-35000 Rennes, France.

[5]University of Chinese Academy of Sciences, Beijing 100049, P. R. China.

[6]Center of Single-Molecule Sciences, Institute of Modern Optics, Frontiers Science Center for New Organic Matter, College of Electronic Information and Optical Engineering, Nankai University, 38 Tongyan Road, Jinnan District, Tianjin 300350, P. R. China.

[†]These authors contributed equally to this work.

*Corresponding authors. E–mail: guoxf@pku.edu.cn (X.G.); smeng@iphy.ac.cn (S.M.); hong.guo@mcgill.ca (H.G.); stephane.rigaut@univ-rennes1.fr (S.R.)

Methods:

Molecular synthesis

Reactions were carried out in an inert atmosphere using Schlenk techniques. Solvents were dried and distilled in an argon atmosphere using standard procedures. ^{1}H, ^{13}C and ^{31}P-NMR spectra were recorded on Bruker 300, 400 and 500 MHz spectrometers in CD_2Cl_2 solutions at 303 K. See Supplementary schemes for the numbering used to assign the spectra. Spectra obtained by high-resolution mass spectrometry (HR-MS) were recorded on a Bruker MicrO-Tof-Q2 spectrometer. The DTE **S1o**[1], compound **S2**[2], **S3**[3] and [ClRu(dppe)$_2$](OTf)[4] were obtained as previously reported.

[(NH$_2$-*p*-C$_6$H$_4$-C≡C)(dppe)$_2$Ru-C≡C-(C$_{15}$S$_2$F$_6$H$_8$)-C≡C-Ru(dppe)$_2$(C≡C-C$_6$H$_4$-*p*-NH$_2$)] **1o:** As shown in Supplementary Scheme 1, in a pre-dried Schlenk tube, the DTE compound **S1o** (100 mg, 0.24 mmol) and [ClRu(dppe)$_2$](OTf) (546 mg, 0.5 mmol) were dissolved in 40 mL of dry dichloromethane and stirred for five days at room temperature. The solvent was then removed under a vacuum and the powder obtained was solubilised in 3 mL of dry dichloromethane. The crude compound **S2** was precipitated by addition of diethylether (15 mL) to afford ~270 mg (~40% yield) of a dark blue powder that was checked via NMR and used immediately without further purification. ^{1}H NMR (400 MHz, CD_2Cl_2): δ(ppm) = 7.39 (m, 16H, H_{ph}), 7.26 (m, 32H, H_{ph}), 7.14 (m, 32H, H_{ph}), 5.76 (s, 2H, H_{thio}), 4.00 (quint., 2H, =CH, $^{4}J_{PH}$ = 3Hz), 2.97–2.74 (br., 16H, PCH$_2$CH$_2$P), 1.47 (s, 6H, CH_3). ^{31}P NMR (162 MHz, CD_2Cl_2): δ(ppm) = 39.61. Then, in a pre-dried Schlenk tube, compound **S3** (30 mg, 0.256 mmol), crude **S2** (270 mg, 0.140 mmol) and $NaPF_6$ (74 mg, 0.436 mmol) were all dissolved in a mixture of 27 mL of dry dichloromethane and 1 mL of Et_3N. The solution was degassed by argon bubbling for 5 min and stirred for six days at room temperature. The solvents were then removed under a vacuum and the powder obtained was subsequently washed sequentially with water (3×8 mL), pentane (3×8 mL) and ether (1×10 mL). Compound **1o** was precipitated purely from an ice-cooled solution of dichloromethane/MeOH (~140 mg, ~50% yield). ^{31}P NMR (162 MHz, CD_2Cl_2): δ(ppm) = 53.08. ^{1}H NMR (400 MHz, CD_2Cl_2): δ(ppm) = 7.76 (m, 16H, H_{ph}), 7.30–7.13 (m, 32H, H_{ph}), 7.00 (m, 32H, H_{ph}), 6.72 (d, $^{3}J_{HH}$ = 8.4 Hz, 4H, H_4), 6.52 (d, $^{3}J_{HH}$ = 8.4 Hz, 4H, H_5), 6.26 (s, 2H, H_6), 2.62 (bs, 16H, H_8), 1.83 (s, 6H, H_7). ^{13}C NMR (101 MHz, CD_2Cl_2) (because the low solubility prevented observation of weak (coupled) signals with complex fluorine coupling overlapping phenyl signals, only the characteristic signals were ascribed unambiguously): δ(ppm) 143.34, 137.95 and 137.42 (m, ipso-Ph (dppe)), 136.15, 135.17 and 134.37 (*o*-Ph (dppe)), 131.17, 130.39, 129.38 and 129.05 (*p*-Ph

(dppe)), 127.60 (*m*-Ph (dppe)), 124.69, 124.44, 121.87, 117.82, 115.16, 107.09 (Ru-C≡C̲-DTE), 32.06 (m, $|^1J_{PC} + {}^3J_{PC}|$ = 22 Hz, PCH_2CH_2P), 14.96 (CH_3) (Supplementary Fig. 1). HR-MS ESI (CH_2Cl_2): *m/z* 2442.4486 $[M]^+$ (calculated 2442.44667 ($C_{139}H_{116}F_6N_2P_8S_2Ru_2$)), 1221.2255 $[M]^{2+}$ (calculated 1221.22306 ($C_{139}H_{116}F_6N_2P_8S_2Ru_2$)). IR (KBr): 2045 ($\nu_{C\equiv C}$) cm^{-1}.

[(C_6H_5-CO-NH-*p*-C_6H_4-C≡C)(dppe)$_2$Ru-C≡C-($C_{15}S_2F_6H_8$)-C≡C-Ru(dppe)$_2$(C≡C-C_6H_4-*p*NH-COC_6H_5)] 2o: As shown in Supplementary Scheme 2, in a pre-dried Schlenk tube, compound **1o** (60 mg, 0.024 mmol), benzoic acid (7.74 mg, 0.061 mmol), 1-ethyl-3-(3-dimethylaminopropyl)carbodiimide (14 mg, 0.073 mmol), and 4-dimethylaminopyridine (7.78 mg, 0.063 mmol) were dissolved in 20 mL of dichloromethane and stirred for 16 h. The solution was then washed with 5×20 mL of water, dried ($MgSO_4$), and filtered off via a cannula into another dry Schlenk tube. Evaporation under reduced pressure produced a dark greyish power. This powder was then washed rigorously with 3×20 mL of degassed methanol and 3×20 mL of pentane to yield ~60 mg (~92%) of compound **2o**.

^{1}H NMR (300 MHz, CD_2Cl_2): δ = 7.88 (d, $^2J_{HH}$ = 6 Hz, 4H, H_3), 7.80 (s, 2H, NH), 7.70 (bd, 16H, H_o), 7.55 (m, 6H, $H_{1,2}$), 7.53 (d, $^2J_{HH}$ = 6 Hz ,4H, H_4), 7.31 (bd, 16H, H_o), 7.22 (m, 16H, H_p), 7.02 (m, 32H, H_m), 6.85 (d, $^2J_{HH}$ = 6 Hz, 4H, H_5), 6.31 (s, 2H, H_6), 2.64 (bs, 16H, H_8), 1.85 (s, 6H, H_7). ^{13}C NMR (126 MHz, CD_2Cl_2): δ 165.30 (C=O), 137.54–137.29 (m, *ipso*-Ph (dppe)), 136.20, 135.84, 134.88 and 134.29 (*o*-Ph (dppe), 132.02, 130.60, 129.34 and 129.16 (*p*-Ph, dppe) 129.04, 127.53 and 127.34 (*m*-Ph, dppe), 124.59, 124.39, 120.10, 113.11 (NHphC̲≡CRu), 107.44 (RuC≡C̲DTE), 31.88 (m, $|^1J_{PC} + {}^3J_{PC}|$ = 24 Hz, CH_2), 14.82 (CH_3). ^{31}P NMR (121 MHz, CD_2Cl_2): δ = 52.96 (s, PCH_2CH_2P). ^{19}F NMR (282 MHz, CD_2Cl_2): δ = −109.8 (4F, F_a), −131.91 (2F, F_b) (Supplementary Fig. 2). IR (KBr): 2033 ($\nu_{C\equiv C}$), 1671 ($\nu_{C=O}$), 1576 ($\delta_{N\text{-}H}$) cm^{-1}.

Isomerisation studies in a macroscopic environment

UV-Vis measurements were performed for compound **2** dissolved in CH_2Cl_2 (1.15×10^{-3} mol·L^{-1}) at 20°C using an Analytik Jena Specord 205 spectrometer. The UV-Vis irradiations were performed in UV cells or in NMR tubes with a LS series light source from ABET Technologies, Inc. (150 W xenon lamp), with single-wavelength light filter numbers 380FS 1025 and 650FS 20-25.

As shown in Supplementary Fig. 9, the bimetallic adduct in CH_2Cl_2 (1.15×10^{-3} mol·L^{-1}) shows an intense absorption band with a high extinction coefficient at λ_{max} = 360 nm, which corresponds to a metal to ligand charge transfer (HOMO ($Ru_{d\text{-}\pi}$) → LUMO (DAE_{π^*})). Upon irradiation at λ_{irr} = 380 nm, this broad band vanishes and another broad band arises in the visible region over a wide range (600–800 nm) characteristic of the closed form of the DAE compound. This means that the d/π(RuC≡C) to π*(DAE) excitation induces sufficient accumulation of density on the two carbon atoms of the DTE to create a single C─C bond upon rotation. It should be noted that, in these irradiation conditions, the closure takes 1 min while re-opening takes 420 min (λ_{irr} = 650 nm), after which ~92% of the initial metal-to-ligand charge transfer (MLCT) band was recovered.

To estimate the photo-stationary state composition, isomerisation was followed by NMR. In the NMR tube, the desired compound **2** was dissolved in CD_2Cl_2 (~10^{-3} mol·L^{-1}) and subjected to illumination at λ_{irr} = 380 nm. The solution colour changed to a dark greenish shade that indicated closure of the compound. After 1 h of irradiation, ^{1}H, ^{31}P, and ^{19}F NMR showed 100% conversion to the closed form with no traces of the open form left. ^{1}H NMR (300 MHz, CD_2Cl_2): δ 7.89 (d, $^2J_{HH}$ = 6 Hz, 4H, H_3), 7.82 (s, 2H, NH), 7.76 (bd, 16H, H_o), 7.55 (m, 6H, $H_{1,2}$), 7.47 (d, $^2J_{HH}$ = 6 Hz ,4H, H_4), 7.25 (m, 16H, H_p), 7.17 (bd, 16H, H_o), 7.05 (m, 32H, H_m), 6.89 (d, $^2J_{HH}$ = 6 Hz, 4H, H_5), 5.21 (s, 2H, H_6), 2.66 (bs, 16H, H_8), 2.16 (s, 6H, H_7). ^{31}P NMR (121 MHz, CD_2Cl_2): δ = 52.28 (s, PCH_2CH_2P). ^{19}F NMR (282 MHz, CD_2Cl_2): δ = −111.0 (d, $^2J_{FaFa'}$ = 262 Hz, $2F_{a/a'}$), −112.8 (d, $^2J_{FaFa'}$ = 262 Hz, $2F_{a/a'}$), −132.62 (2F, F_b). See Supplementary Fig. 3. Note that the procedures of molecular synthesis are reprinted with permission from Ref. 8. Copyright 2021 American Chemical Society.

Analysis of single-molecule connection

As shown elsewhere[5,6], the number of junctions that contribute to the charge transport can be determined by calculating the probability of connected devices with *n*–rejoined junctions (G_n) with a binomial distribution and an optimised connection, which yields:

$$G_n = \frac{m!}{n!(m-n)!} p^n (1-p)^{m-n} \quad n = 0, 1, 2 \cdots, m \tag{1}$$

where m is the number of graphene point contact pairs (210 in the current case) and p is the probability of successful connection for a random junction. The possibility of a connected junction γ_c can therefore be attained as:

$$\gamma_c = 1 - G_0 = 1 - \frac{m!}{0!(m-0)!} p^0 (1-p)^m = 1 - (1-p)^m \qquad (2)$$

where G_0 is the probability of devices without any connected junctions. In our experiments, ~5% of the junctions show successful connection to molecules and the corresponding possibility of successful connection for each contact pair (p) is $0.05/m$. The ratio of single–junction devices to the overall number of reconnected devices is then ~97.5%. These results from calculations suggest that, in most cases, charge transport in these devices arises mainly in a single–molecule junction. Further experimental evidence for the successful formation of GMG-SMJs can be found in a recent work[7], where we used a self-built photoelectrical integrated characterization system to demonstrate the single-molecule connection.

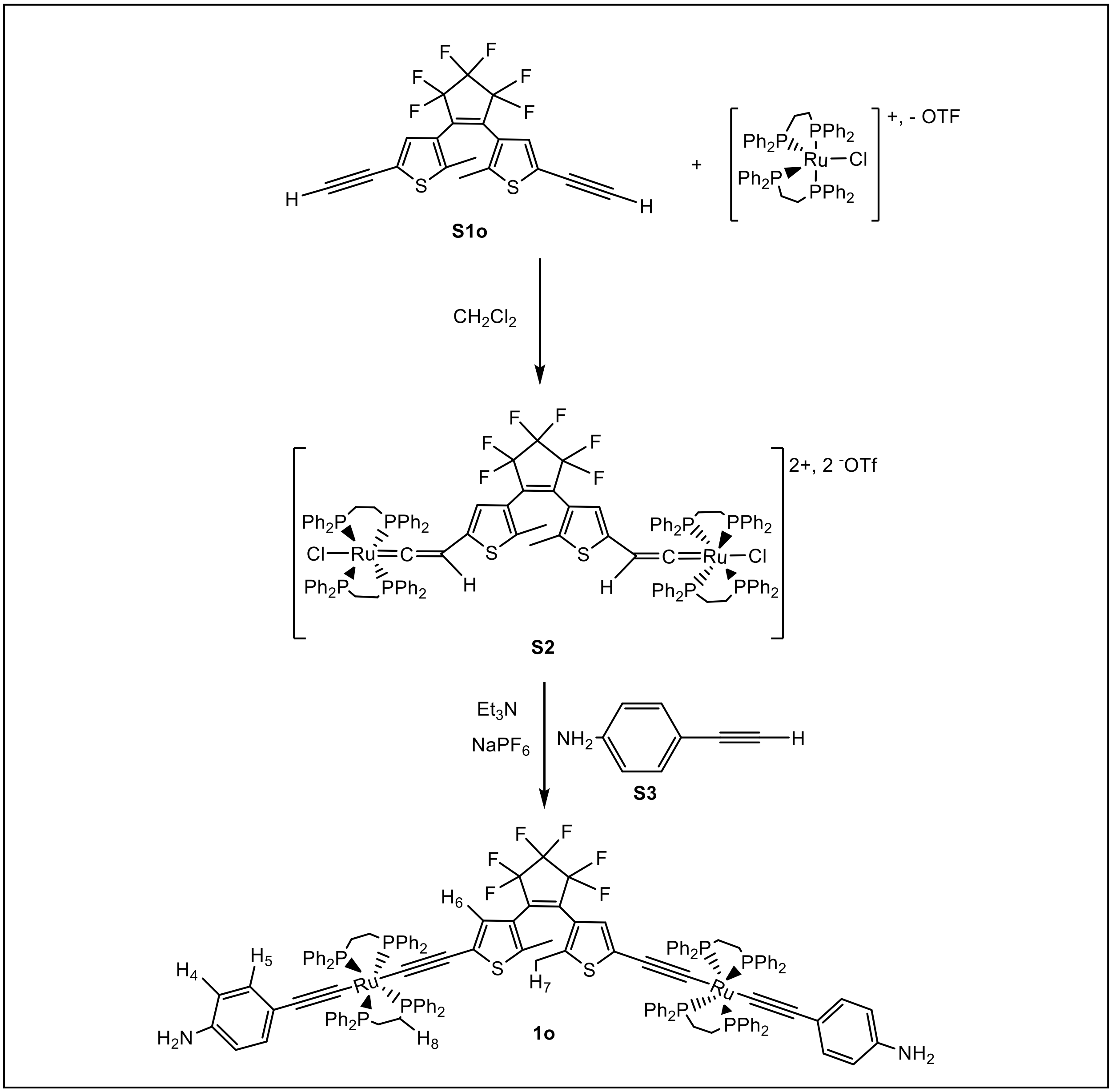

Supplementary Scheme 1 | Synthesis of complex 1o. Amino groups on both ends of Ru-DAE were used to react with carboxylic groups at the edges of graphene point contacts to form covalent amide bonds, thus building stable single-molecule junctions. Reprinted with permission from Ref. 8. Copyright 2021 American Chemical Society.

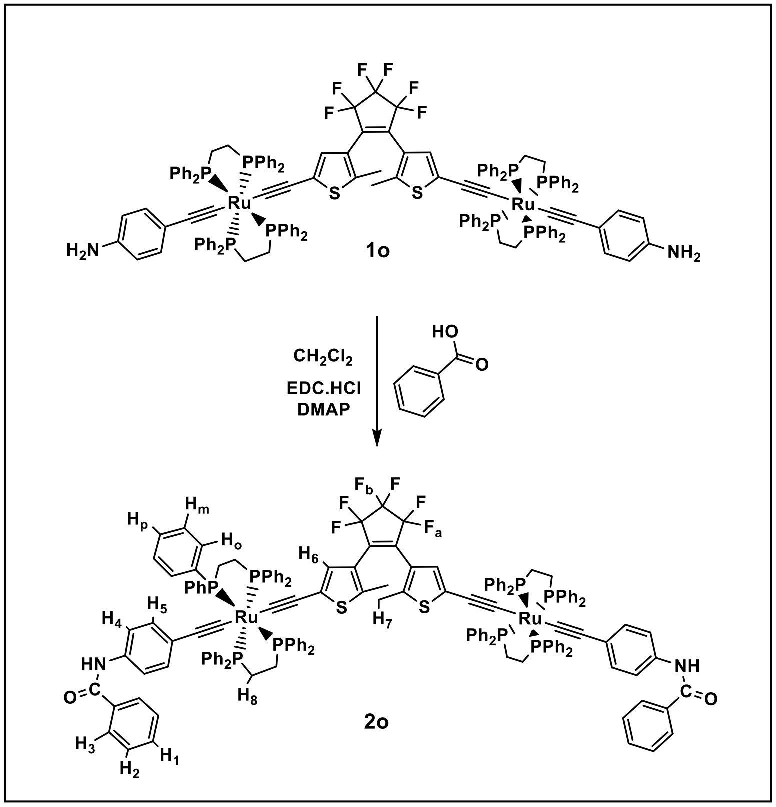

Supplementary Scheme 2 | Synthesis of complex 2o. Reprinted with permission from Ref. 8. Copyright 2021 American Chemical Society.

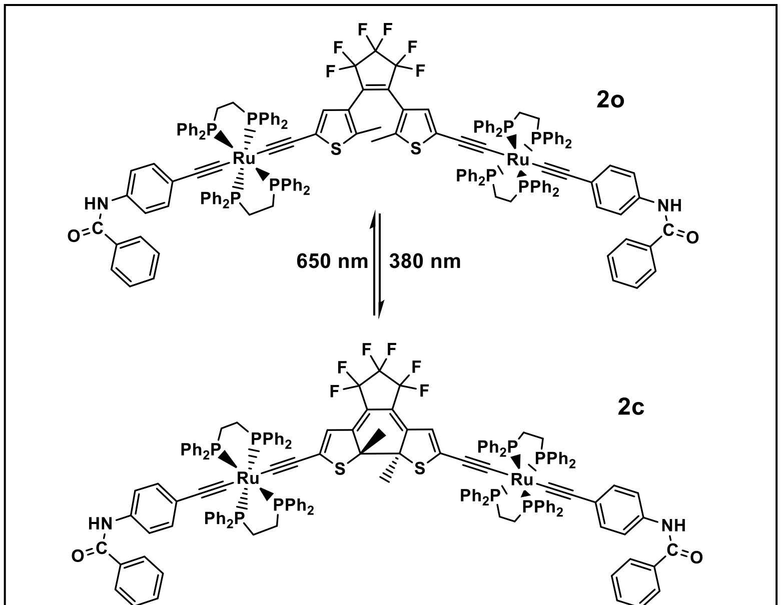

Supplementary Scheme 3 | Illustration of isomerisation for 2o. Reprinted with permission from Ref. 8. Copyright 2021 American Chemical Society.

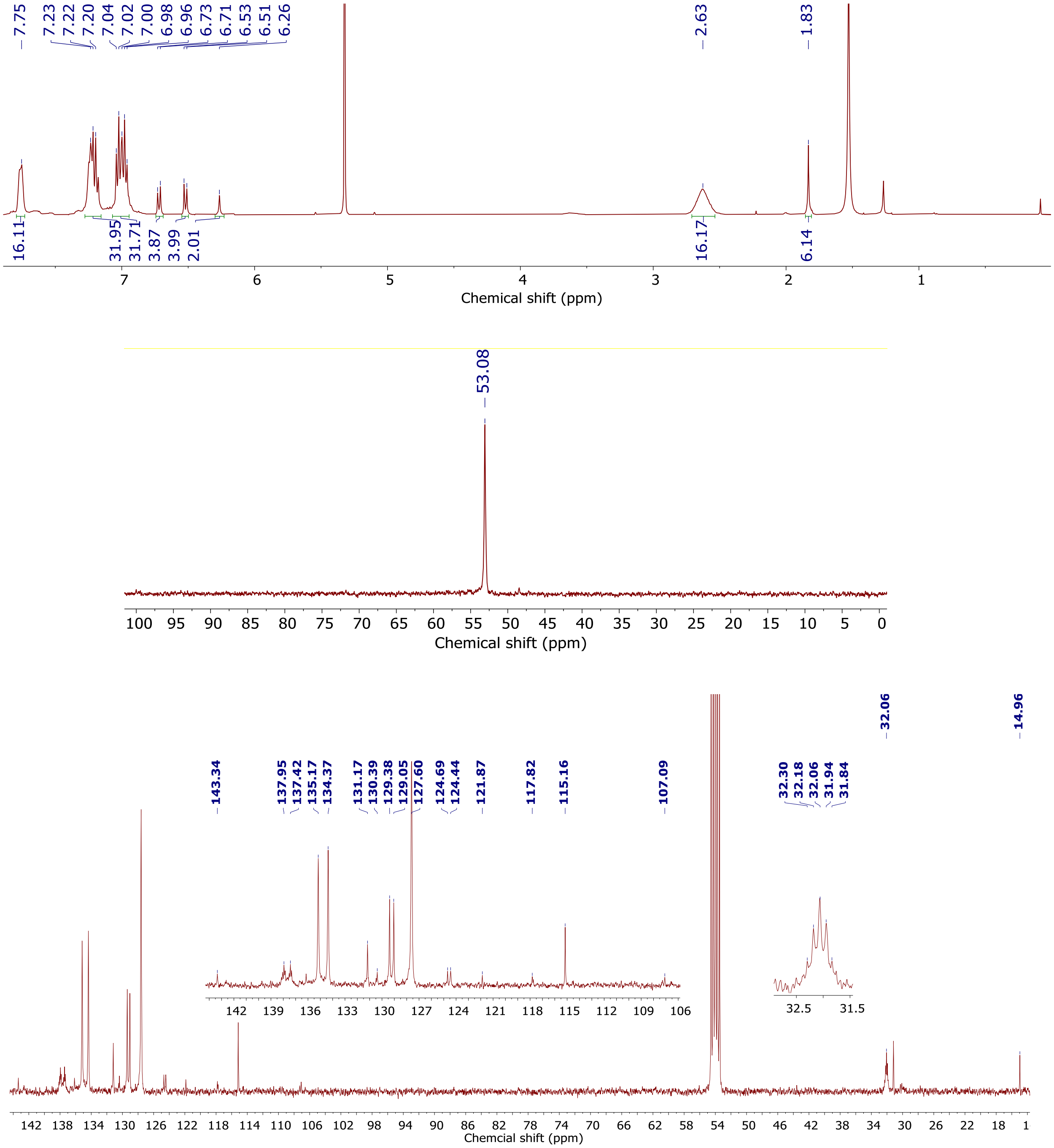

Supplementary Fig. 1 | ^{1}H (top),^{31}P (middle), and ^{13}C (bottom) NMR spectra of 1o in CD_2Cl_2.
Reprinted with permission from Ref. 8. Copyright 2021 American Chemical Society.

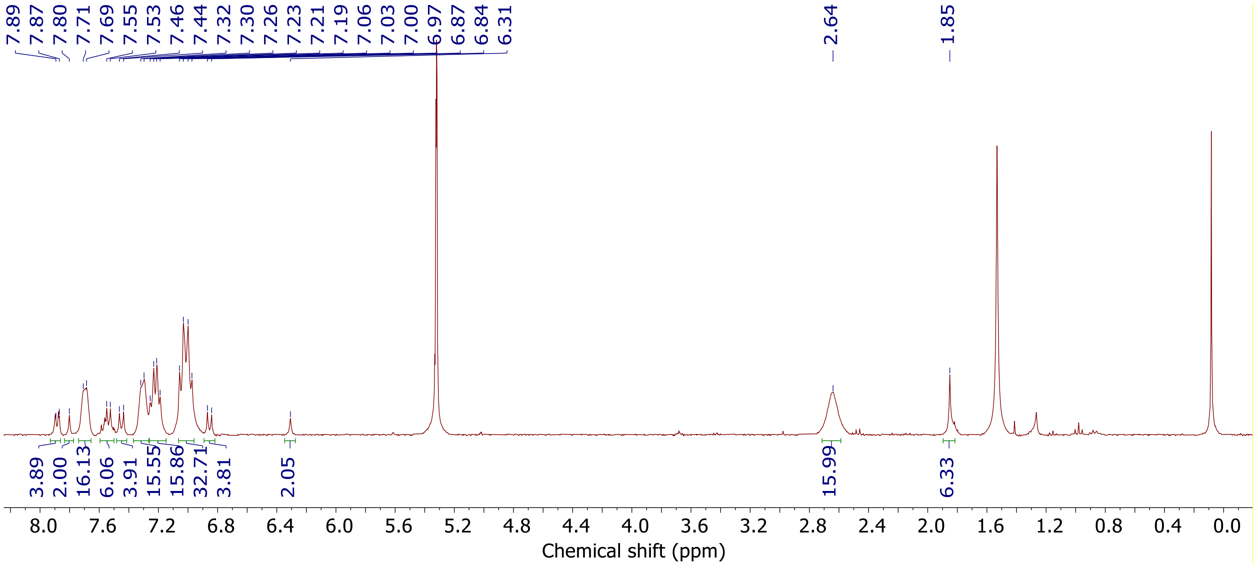
7.89 7.87 7.80 7.71 7.69 7.55 7.53 7.46 7.44 7.32 7.30 7.26 7.23 7.21 7.19 7.06 7.03 7.00 6.97 6.87 6.84 6.31
2.64
1.85
3.89 2.00 16.13 6.06 3.91 15.55 15.86 32.71 3.81 2.05
15.99
6.33
8.0 7.6 7.2 6.8 6.4 6.0 5.6 5.2 4.8 4.4 4.0 3.6 3.2 2.8 2.4 2.0 1.6 1.2 0.8 0.4 0.0
Chemical shift (ppm)

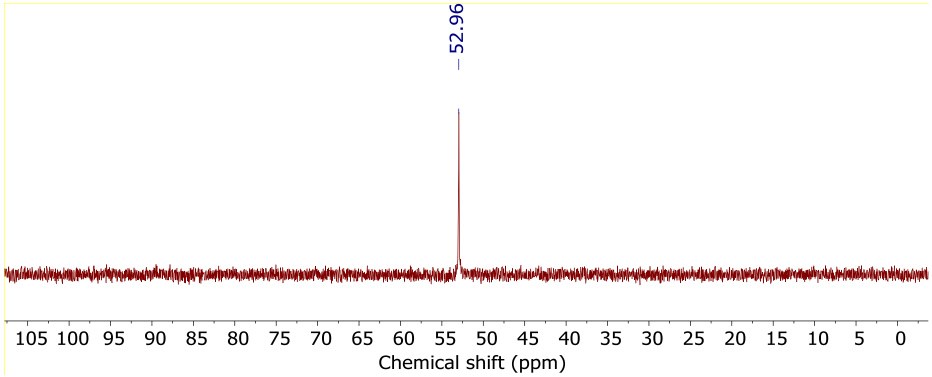
52.96
105 100 95 90 85 80 75 70 65 60 55 50 45 40 35 30 25 20 15 10 5 0
Chemical shift (ppm)

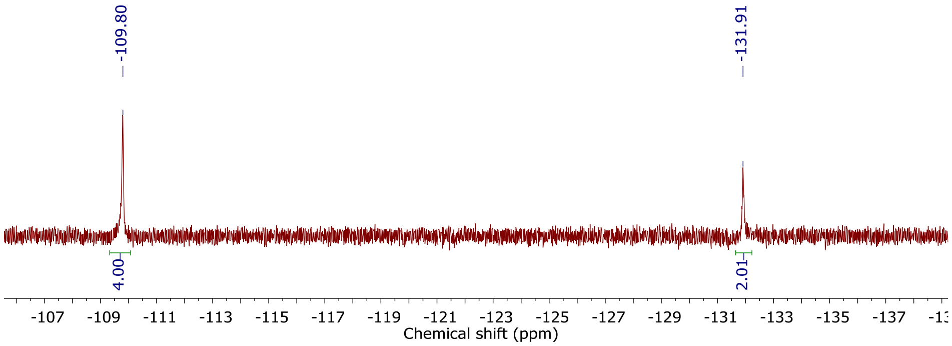
-109.80
-131.91
4.00
2.01
-107 -109 -111 -113 -115 -117 -119 -121 -123 -125 -127 -129 -131 -133 -135 -137
Chemical shift (ppm)

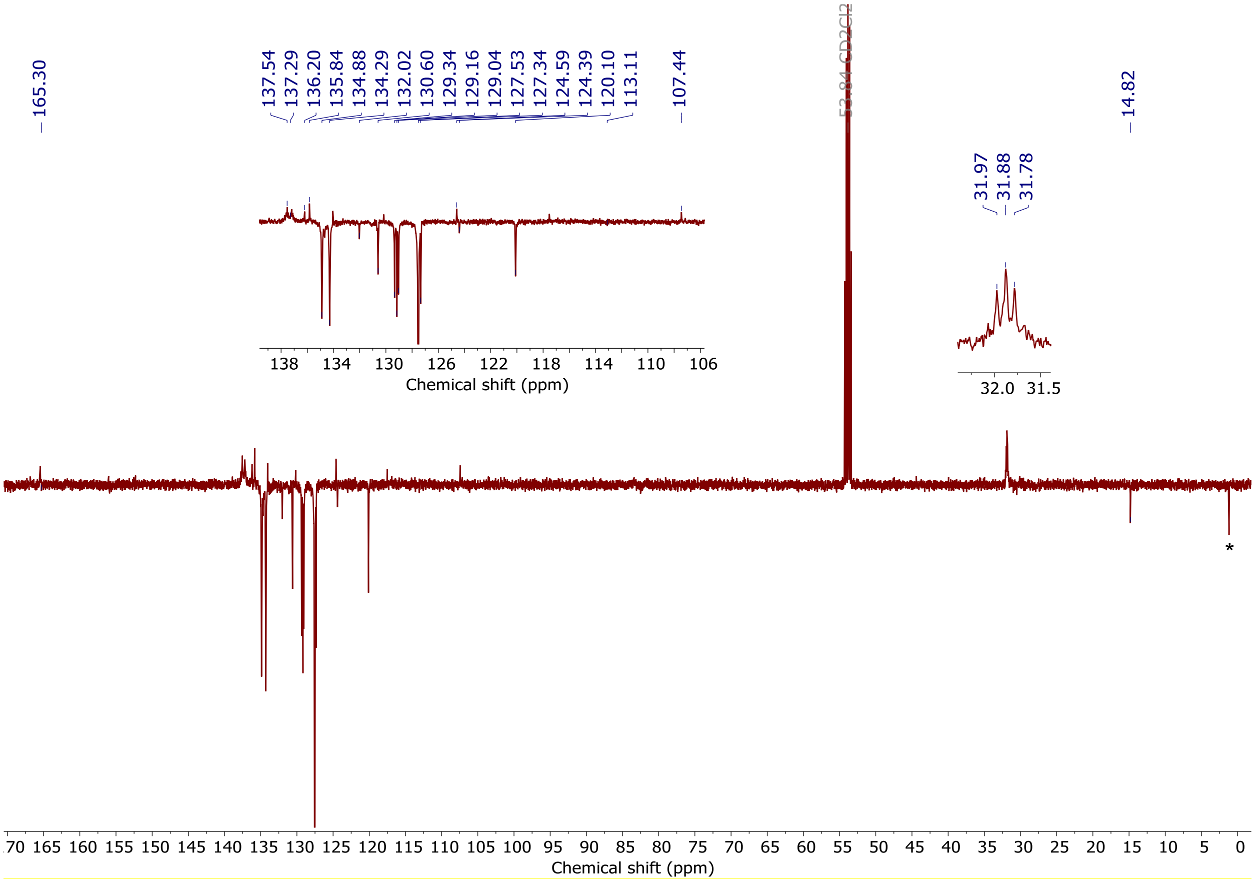

Supplementary Fig. 2 | ^{1}H, ^{31}P, ^{19}F and ^{13}C (dept-135) (from top to bottom) NMR spectra of 2o in CD_2Cl_2. Reprinted with permission from Ref. 8. Copyright 2021 American Chemical Society.

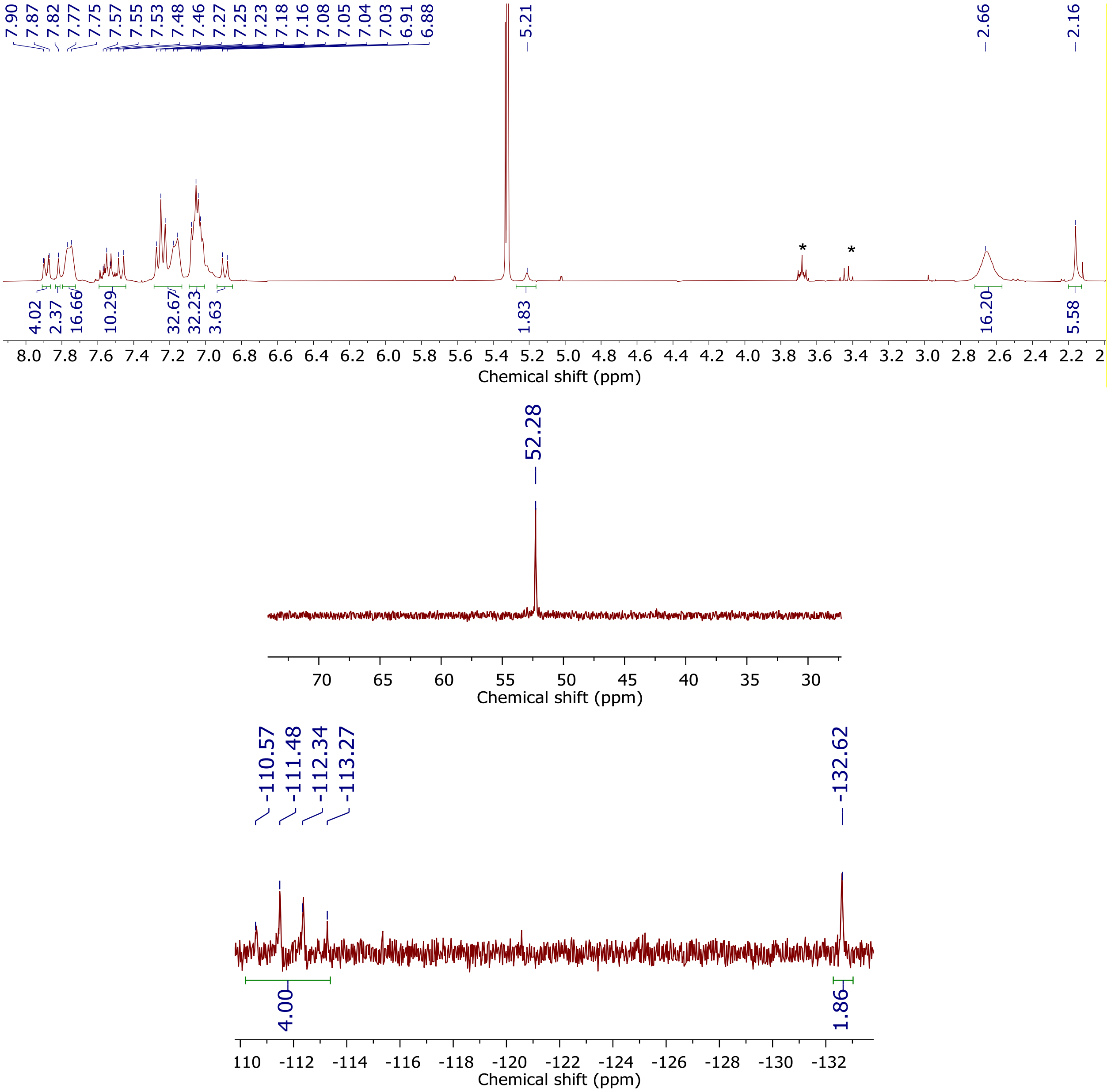

Supplementary Fig. 3 | ^{1}H, ^{31}P and ^{19}F (from top to bottom) NMR spectra of 2c (PSS after 380 nm irradiation) in CD_2Cl_2. Reprinted with permission from Ref. 8. Copyright 2021 American Chemical Society.

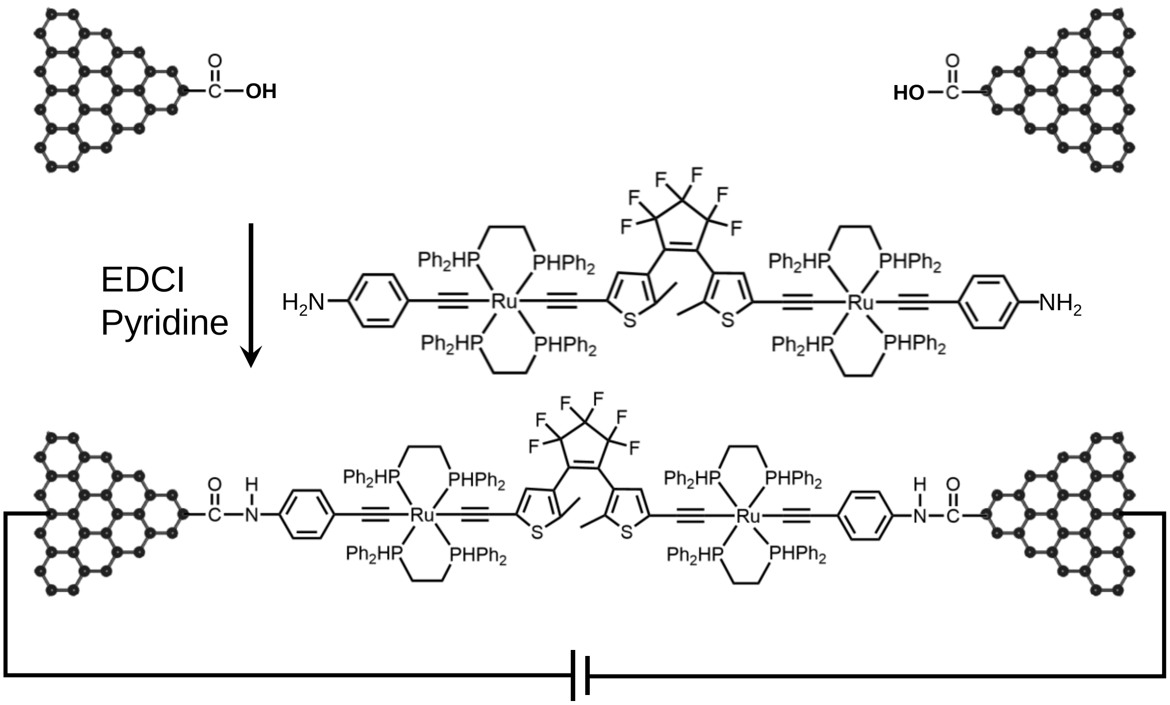

Supplementary Fig. 4 | The procedure to connect individual diarylethenes with graphene electrodes.

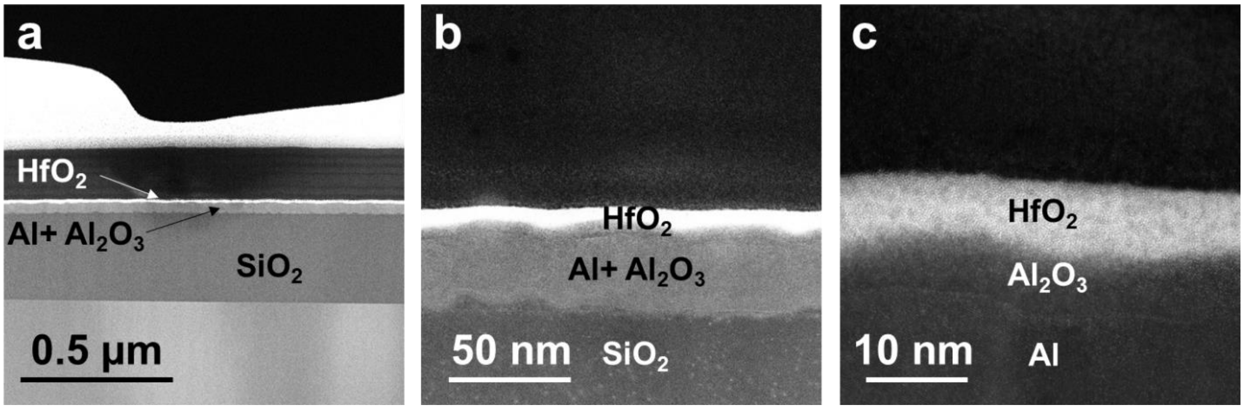

Supplementary Fig. 5 | STEM images of the cross section. The STEM images show the dielectric structure with ~5 nm Al_2O_3 and ~5 nm HfO_2. The roughness is improved by the deposition of HfO_2 with a sol-gel method.

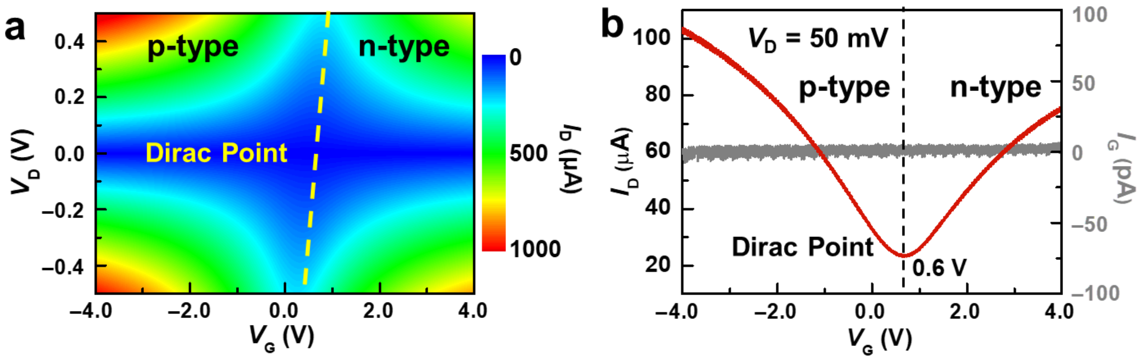

Supplementary Fig. 6 | Transport characteristics of a pristine graphene ribbon. (**a**) Two-dimensional visualizations of conductance (I_D) plotted versus V_G and V_D. The absolute current value is used. The white dashed line is the position of the Dirac point. (**b**) Transfer curve (I_D–V_G) at a fixed bias voltage of 50 mV. The gray line is the gate leakage (I_G) on the right axis.

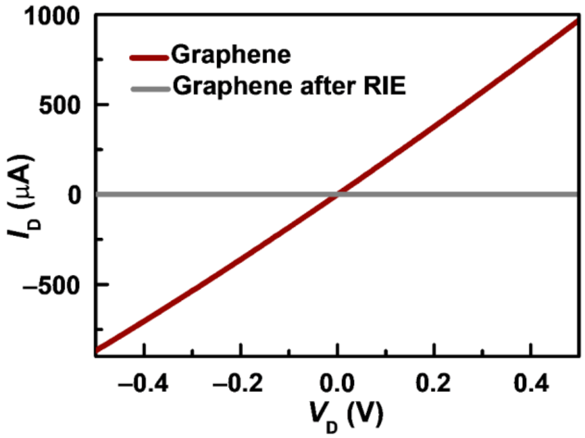

Supplementary Fig. 7 | *I*–*V* curves before (red) and after (grey) oxygen plasma etching. The gate voltage is 0 V.

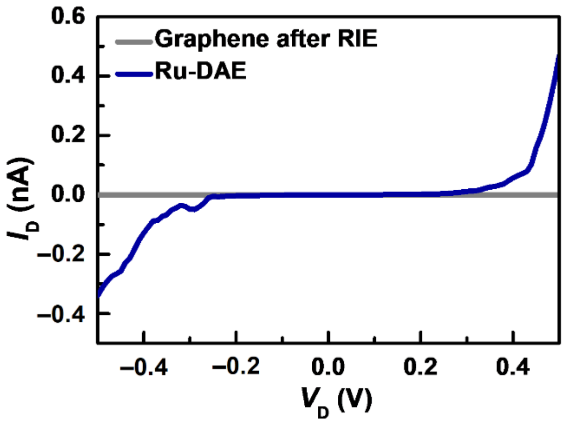

Supplementary Fig. 8 | *I*–*V* curves of a device at different stages: nanogapped graphene (grey solid line) and Ru-DAE-connected graphene (blue solid line). The gate voltage is 0 V.

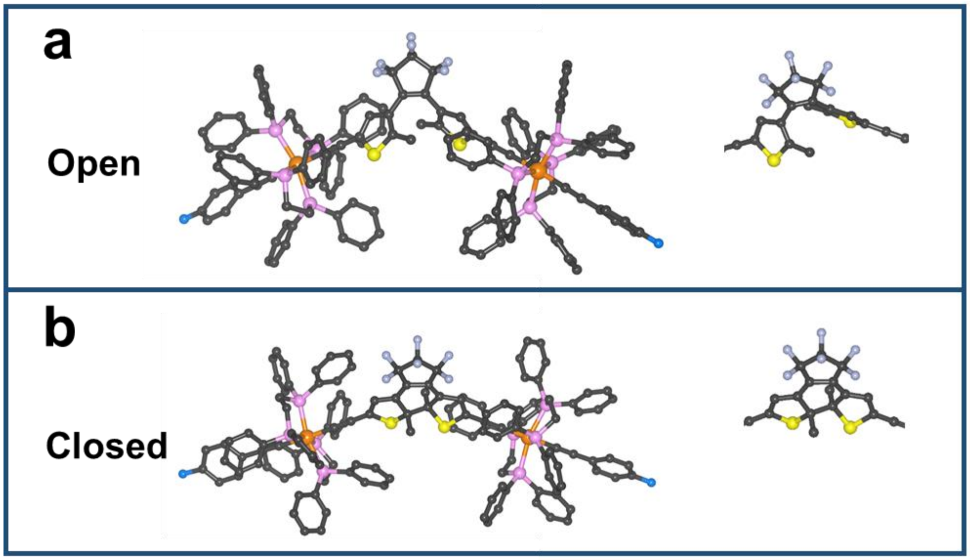

Supplementary Fig. 9 | Relaxed structures of Ru-DAE complexes with ring-open (a) and ring-closed (b) states. The right panel is the highlight of the diarylethene part.

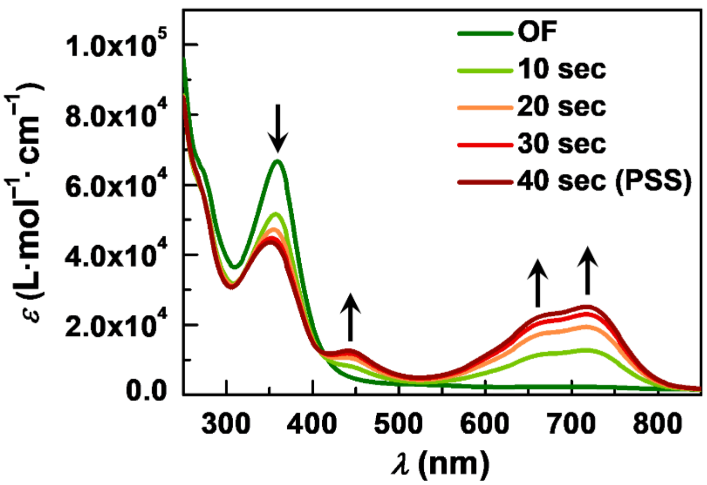

Supplementary Fig. 10 | Time-dependent UV-Vis absorption spectra of 2o under UV irradiation. The bimetallic adduct displays in CH_2Cl_2 (1.15×10^{-3} mol·L^{-1}) an intense absorption band with a large extinction coefficient at λ_{max} = 360 nm, which corresponds to metal-to-ligand charge transfer (MLCT) (HOMO ($Ru_{d\text{-}\pi}$) → LUMO ($DAE_{\pi*}$)). Upon irradiation with λ_{irr} = 380 nm, this broad band decreases and a broad band rises in the visible region over a wide range (600–800 nm), which corresponds to the closed form of the DAE compound. This means that the d/π(RuC≡C) to π*(DAE) excitation induces enough accumulation of density on two carbon atoms of the DTE for the creation of a single C–C bond upon rotation. It is worth noting that, in these conditions, the closure takes 1 minute while re-opening takes 420 minutes (λ_{irr} = 650 nm) and ~92% of the initial MLCT was recovered. Reprinted with permission from Ref. 8.

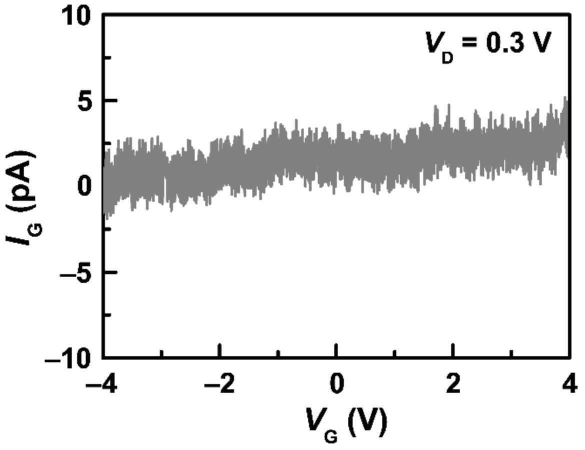

Supplementary Fig. 11 | I_G–V_G curves of the device in Fig. 3. The leakage current is negligible.

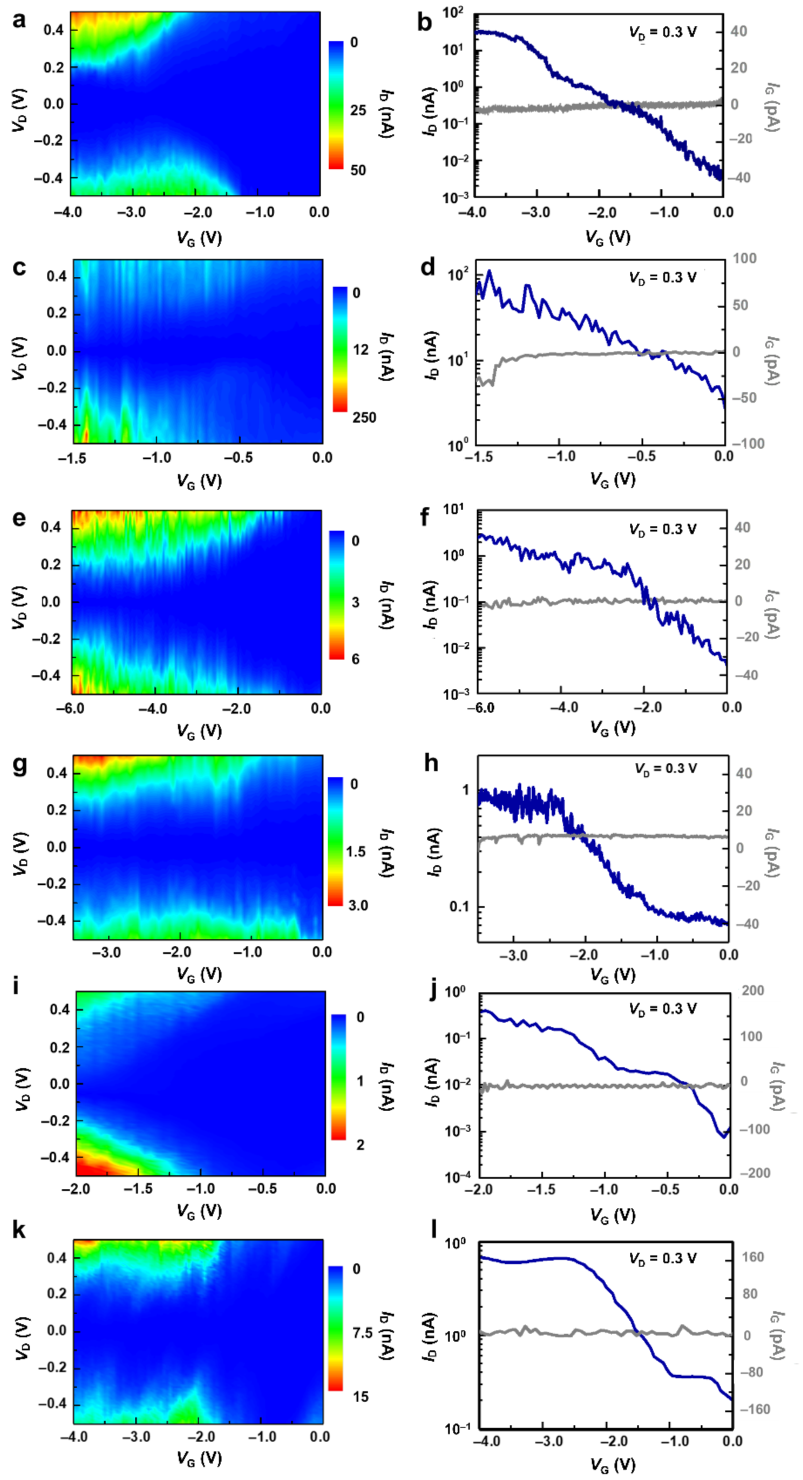

Supplementary Fig. 12 | Device characteristics of different Ru-*o*DAE single-molecule FETs. (**a, c, e, g, i and k**) Two-dimensional visualizations of conductance (I_D) plotted versus V_G and V_D. The absolute current value is used. (**b, d, f, h, j and l**) Transfer curves with logarithm coordinate at a fixed V_D of 0.3 V. In addition, the current mappings of all the devices also demonstrate the rectification behavior due to gate-induced symmetry breaking, which has been studied in detail in our other work.

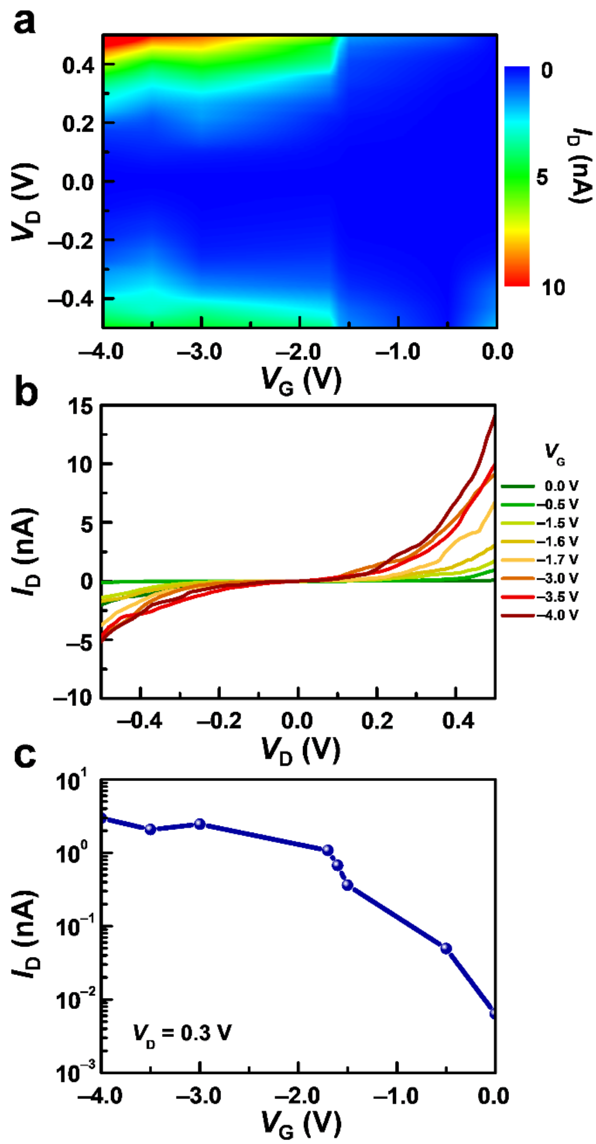

Supplementary Fig. 13 | Gate-controllable charge transport in another Ru-*o*DAE single-molecule transistor. (**a**) Two-dimensional visualization of I_D *vs*. V_G and V_D, interpolated from nine current-voltage curves under different gate voltages. (**b**) Representative I_D–V_D curves for different values of V_G. (**c**) Transfer characteristics for the Ru-*o*DAE single-molecule FET at V_D = 0.3 V.

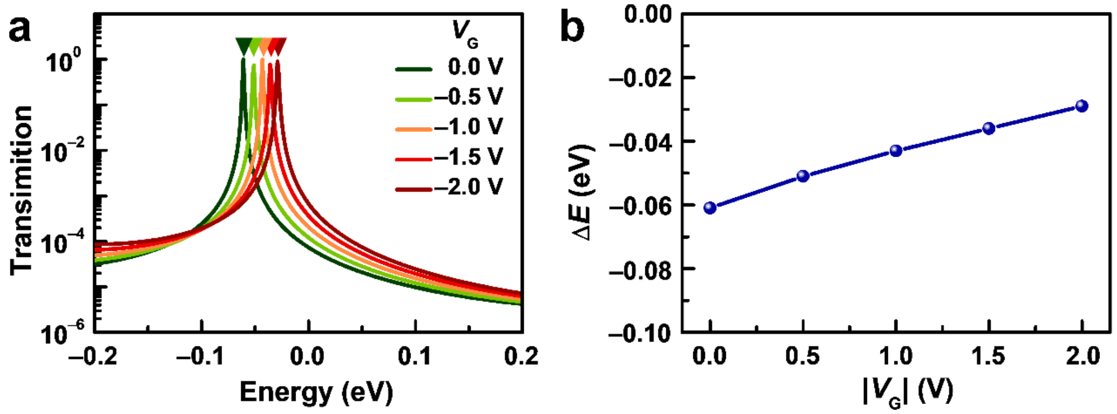

Supplementary Fig. 14 | Calculated transmission spectra and *p*-HOMO position of Ru-*c*DAE tuned by the gate voltage. The zero position of energy in **(a)** and **(b)** are the Fermi level position of graphene.

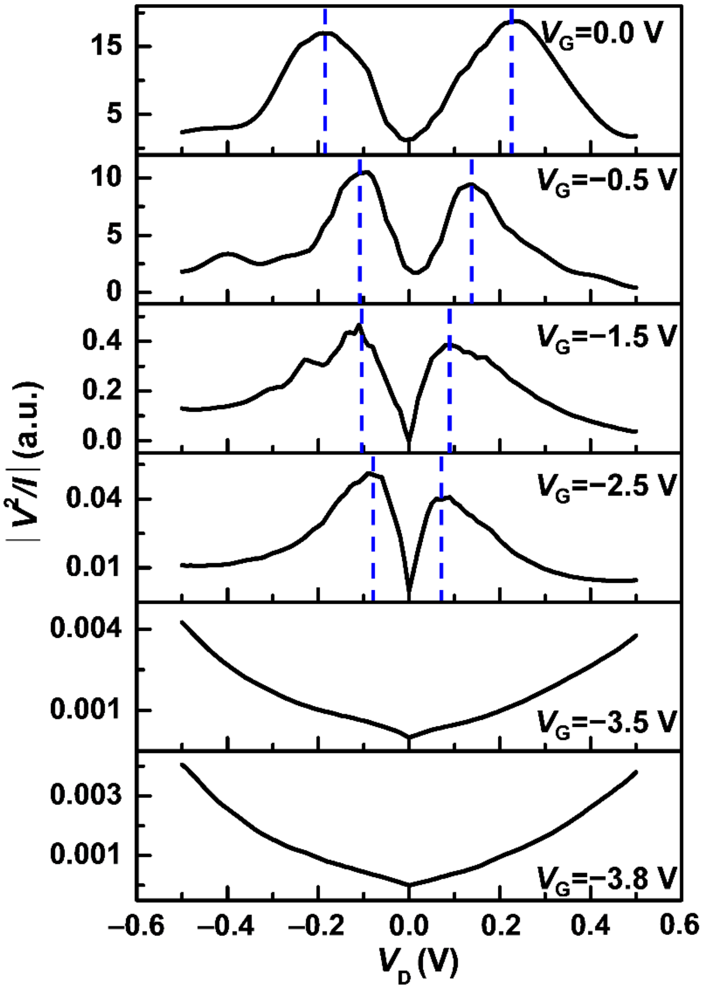

Supplementary Fig. 15 | $|V^2/I|$ *vs.* V curves at different gate voltages for the device in Fig. 3 (Ru-oDAE). The HOMO falls in the Fermi level as the gate voltage goes to −3.5 V.

Supplementary Table 1 | The "natural" units V_C and I_C for different gate voltages in Supplementary Fig. 15.

V_G	V_C_negative	V_C_positive	V_C	I_C_negative	I_C_positive	I_C
**−0.0V**	−0.191	0.231	0.211	−0.002144	0.00286	0.002502
**−0.5V**	−0.115	0.149	0.132	−0.001259	0.002356	0.001808
**−1.5V**	−0.110	0.092	0.101	−0.02728	0.02194	0.02461
**−2.5V**	−0.088	0.073	0.081	−0.1518	0.1319	0.1419

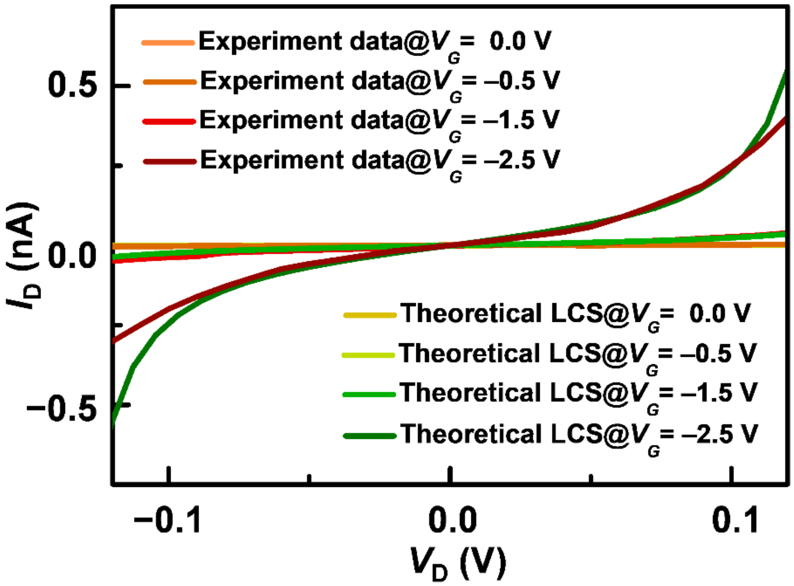

Supplementary Fig. 16 | Experimental and theoretical *I–V* curves for different gate voltages, where theoretically $I = I_R{*}I_C$ and $V = V_R{*}V_C$. Based on the law of corresponding states (LCS) for electron tunneling mediated by a single level in molecular junctions, the maxima of $|V^2/I|$ *vs.* V curves can be used to define "natural" units V_C and I_C for voltage and current, respectively, as shown in Supplementary Table 1. The theoretical *I–V* curves are based on the relationship of dimensionless biases ($V \equiv V_R{*}V_C$) and currents ($I \equiv I_R{*}I_C$), where I_R and V_R can be expressed as follows:

$$I_R = \frac{2V_R}{3 - V_R^2}$$

The theoretical gate dependent *I–V* curves are achieved based on the differences of V_C and I_C in Supplementary Table 1, which vary along with the gate voltage.

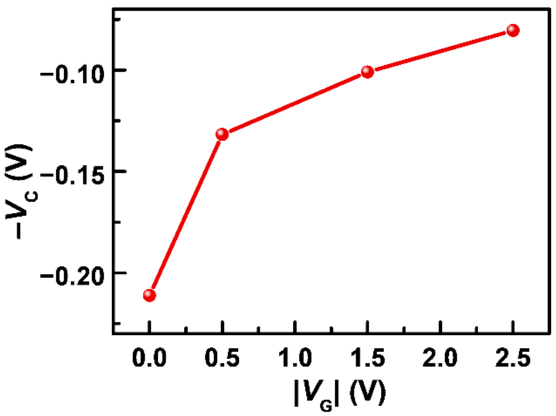

Supplementary Fig. 17 | The peak bias in Supplementary Fig. 15 relative to the gate voltage. The energy gap between *p*-HOMO and the Fermi level of graphene electrodes decreases as the gate voltage becomes more negative.

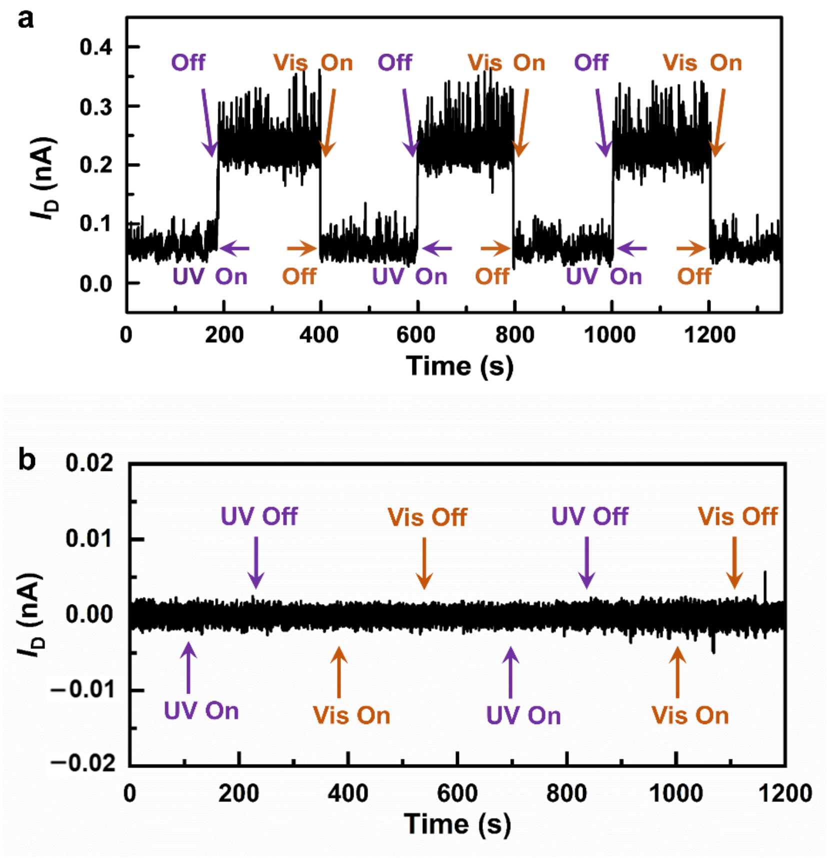

Supplementary Fig. 18 | Reversible photoswitching of another graphene-Ru-DAE-graphene single-molecule junction. (**a**) Real-time measurement of the current through a diarylethene molecule that reversibly switches between ring-closed and ring-open forms upon exposure to alternate ultraviolet (UV: 380 nm) and visible (Vis: 650 nm) irradiations. V_D = 100 mV and V_G = 0 V. (**b**) Control experiments showing the absence of optical switching prior to molecules deposition. V_D = 100 mV and V_G = 0 V.

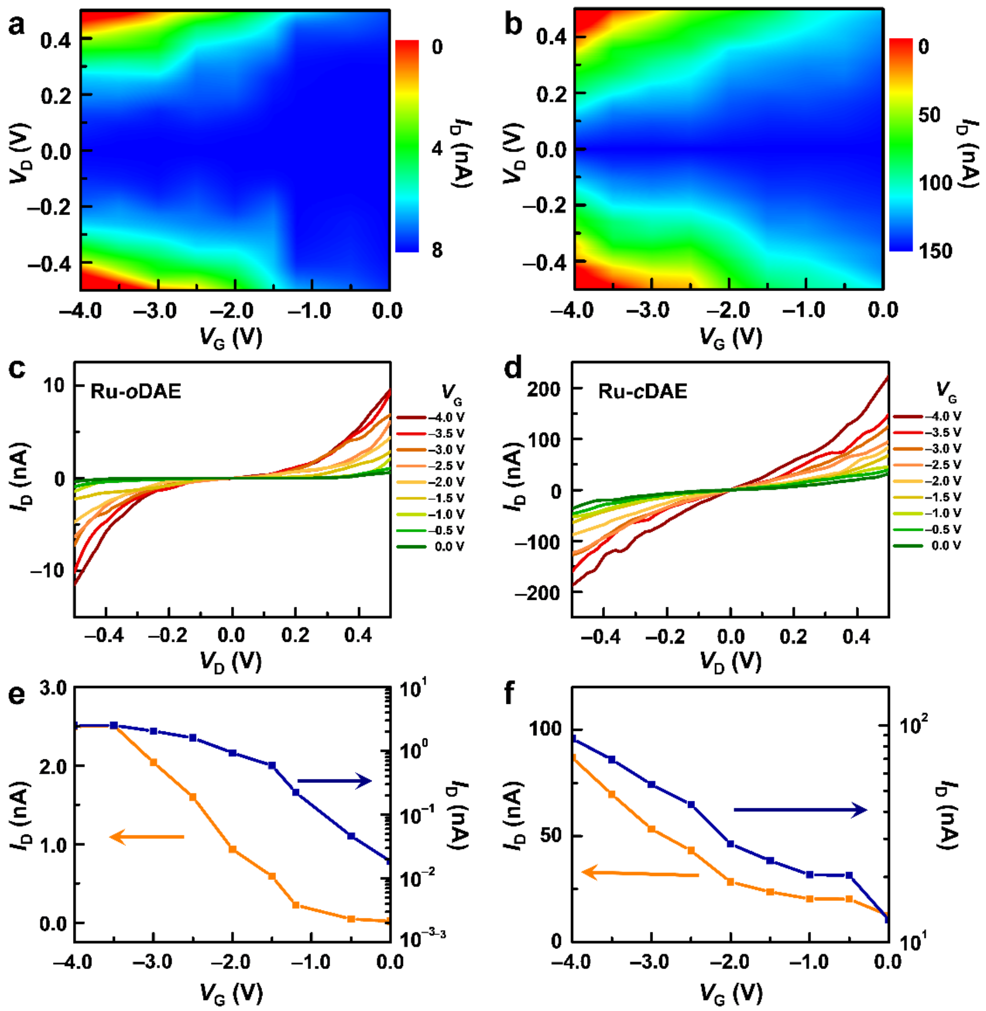

Supplementary Fig. 19 | The gating effect of ring-open and ring-closed states for the same device in Fig. 2a. (**a, c and e**) are under visible irradiation and (**b, d and f**) are after UV irradiation. (**a** and **b**) Two-dimensional visualization of I_D vs. V_G and V_D, interpolated from the current-voltage characteristics under different V_G with interval of 500 mV. (**c** and **d**) Representative I_D vs. V_D curves for different values of V_G. (**e** and **f**) Corresponding transfer characteristics at V_D = 300 mV.

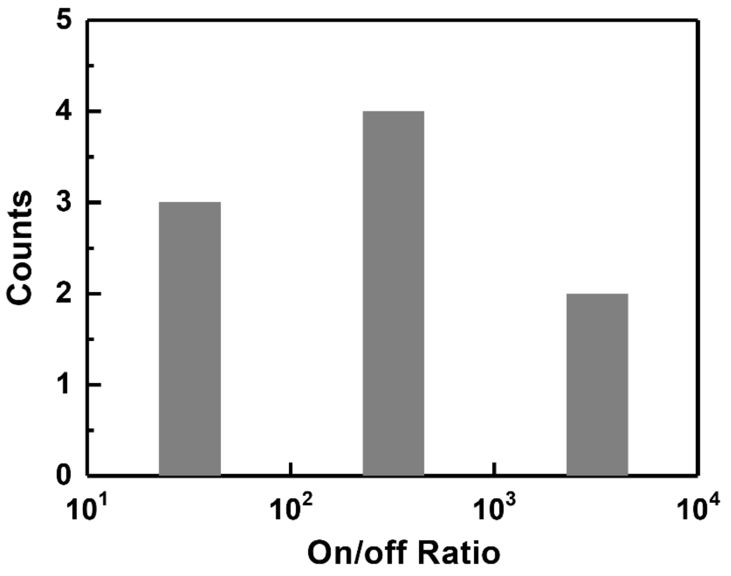

Supplementary Fig. 20 | Statistic distribution of on/off ratios among different devices in the ring-open state at V_D = 300 mV.

References

1. Osuka, A., Fujikane, D., Shinmori, H., Kobatake, S. & Irie, M. Synthesis and photoisomerization of dithienylethene-bridged diporphyrins. *J. Org. Chem.* **66**, 3913–3923 (2001).
2. Liu, Y., Lagrost, C., Costuas, K., Tchouar, N., Bozec, H. L. & Rigaut, S. A multifunctional organometallic switch with carbon-rich ruthenium and diarylethene units. *Chem. Commun.* 6117–6119 (2008).
3. Zhang, J. *et al.* Design, synthesis, and structure-activity relationship of 7-propanamide benzoxaboroles as potent anticancer agents. *J. Med. Chem.* **62**, 6765–6784 (2019).
4. Fox, M. A. *et al.* A simple synthesis of *trans*-RuCl(C≡CR)(dppe)$_2$ complexes and representative molecular structures. *J. Organomet. Chem.* **694**, 2350–2358 (2009).
5. Jia, C., Wang, J., Yao, C., Cao, Y., Zhong, Y., Liu, Z., Liu, Z., Guo, X. Conductance switching and mechanisms in single-molecule junctions. *Angew. Chem. Int. Ed.* **52,** 8666–8670 (2013).
6. Meng, L. *et al.* Side-group chemical gating via reversible optical and electric control in a single molecule transistor. *Nat. Commun.* **10**, 1450 (2019).
7. Yang, C., *et al.* Electric field–catalyzed single-molecule Diels-Alder reaction dynamics. *Sci. Adv.* **7**, eabf0689 (2021).
8. Xin, N., *et al.* Tunnable symmertry-breaking-induced dual functions in stable and photoswitched single-molecule junctions. *J. Am. Chem. Soc.* **143**, 20811–20817 (2021).
